# Supplementary material for: Evaluation of Aspergillus niger Six Constitutive Strong Promoters by Fluorescent-Auxotrophic Selection Coupled with Flow Cytometry: A Case for Citric Acid Production
Source: J Fungi (Basel). 2022 May 26;8(6):568. doi: 10.3390/jof8060568 (PMC9224621; doi:10.3390/jof8060568)
Supplement: Supplementary file 1 [file jof-08-00568-s001.zip › jof-1706358-supplementary.pdf]

Supporting Information

Evaluation of *Aspergillus niger* Six Constitutive Strong Promoters by Fluorescent-Auxotrophic Selection Coupled with Flow Cytometry: A Case for Citric Acid Production

Yudan Lu [1,2,3,†], Xiaomei Zheng [2,3,4,5,†], Yu Wang [2,3,4,5], Lihui Zhang [2], Lixian Wang [2,5], Yu Lei [2,5], Tongcun Zhang [1], Ping Zheng [2,3,4,5,*] and Jibin Sun [2,3,4,5,*]

1 College of Biotechnology, Tianjin University of Science & Technology, Tianjin 300457, China; luyd@tib.cas.cn (Y.L.); zhangtc@tust.edu.cn (T.Z.)
2 Tianjin Institute of Industrial Biotechnology, Chinese Academy of Sciences, Tianjin 300308, China; zheng_xm@tib.cas.cn (X.Z.); wang_y@tib.cas.cn (Y.W.); zhang_lihui@126.com (L.Z.); wang_lx@tib.cas.cn (L.W.); lei_y@tib.cas.cn (Y.L.)
3 Key Laboratory of Systems Microbial Biotechnology, Chinese Academy of Sciences, Tianjin 300308, China
4 College of Life Sciences, University of Chinese Academy of Sciences, Beijing 100049, China
5 National Technology Innovation Center of Synthetic Biology, Tianjin 300308, China
* Correspondence: zheng_p@tib.cas.cn (P.Z.); sun_jb@tib.cas.cn (J.S.)
† These authors contributed equally to this work.

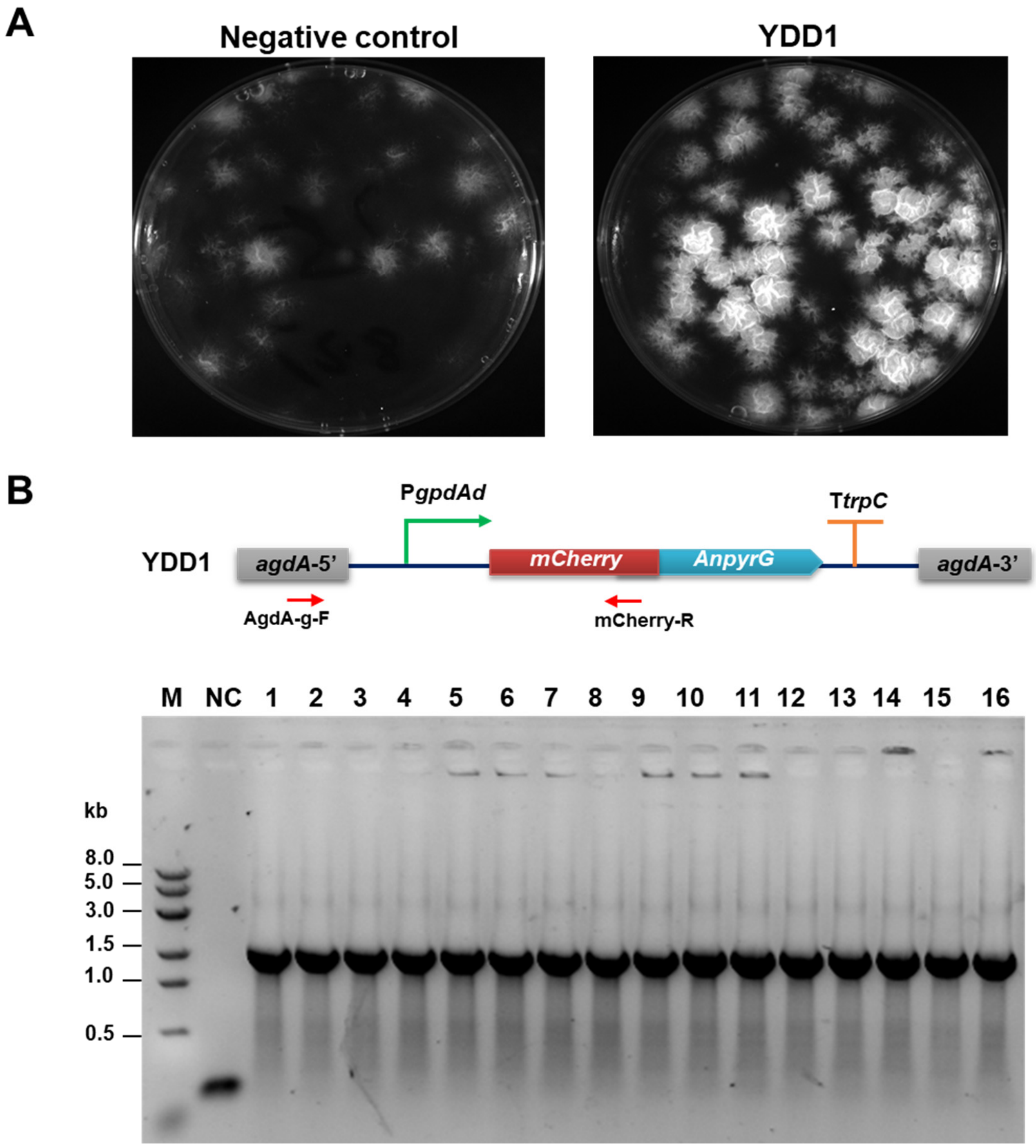

Figure S1. Construction of *A. niger* strain expressing *mCherry-pyrG* under the control of the P*gpdAd* promoter.

(A) Fluorescence image acquisition of primary transformants using Tanon fluorescence camera system. YDD1 represents the primary transformants co-transformed with sgRNAs, pCas9 and donor DNAs harboring P*gpdAd*::*mCherry-pyrG* expressing cassette, leading to potential positive transformants with significant fluorescence. In contrast, the negative control only transformed with pCas9-AnpyrG, resulting in the transformants without significant fluorescence. (B) Diagnostic PCR analysis of YDD1 transformants with fluorescence. The

expected sizes of PCR products were 1549-bp (agdA-g-F/mCherry-R), when the donor DNAs harboring P*gpdAd*::*mCherry-pyrG* expressing cassette was correctly integrated into the *agdA* locus. The parent strain D353.8 was used as negative control, which was represented as “NC” in each electrophoretogram. YDD1.2, YDD1.6, YDD1.9 and YDD1.13 were selected for further quantitative fluorescence detection.

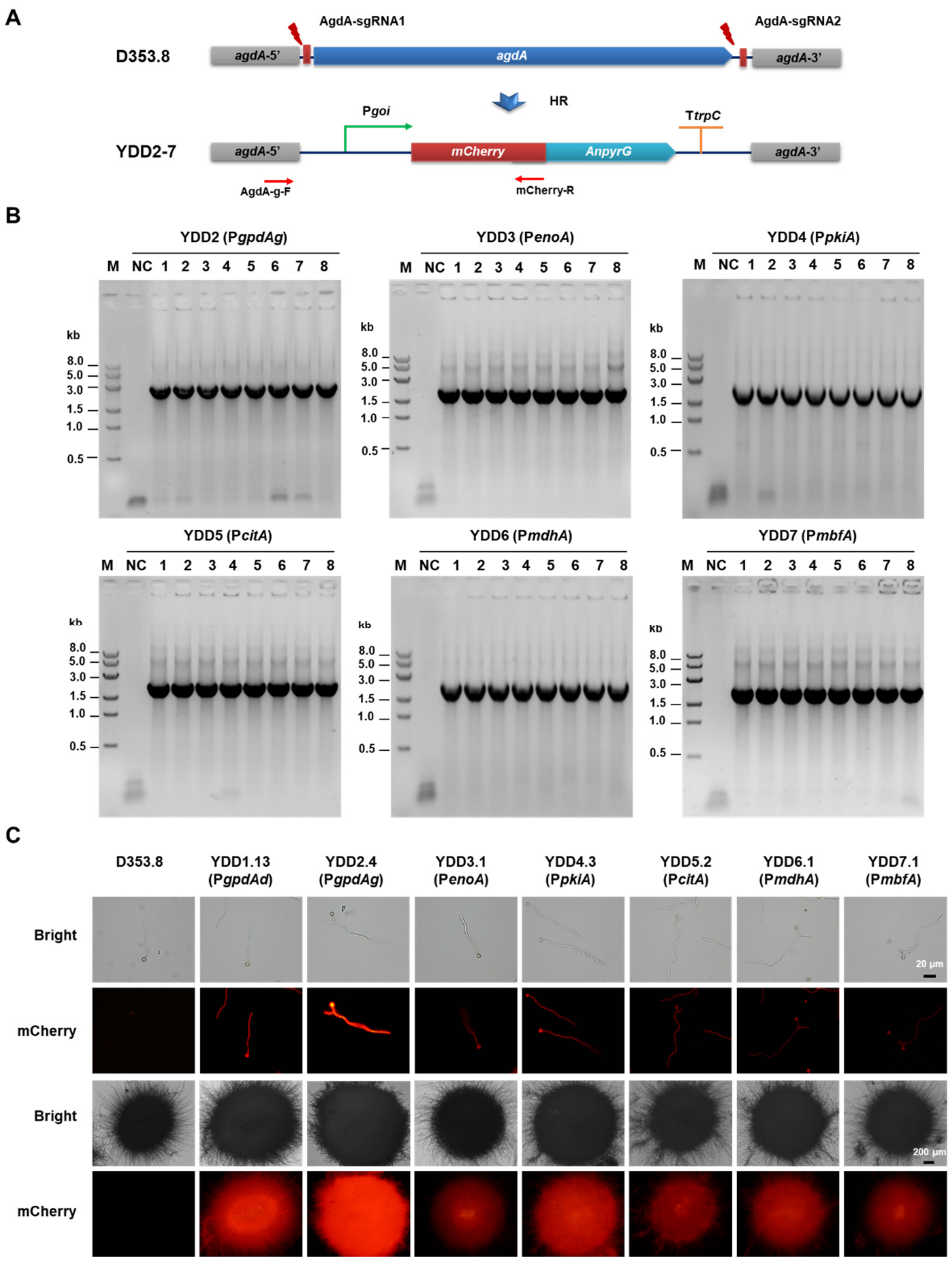

Figure S2. Constructs expressing *mCherry-pyrG* controlled by six constitutive promoters. (A) Schematic diagram of constructs expressing *mCherry-pyrG* controlled by six constitutive promoters. The donor DNAs were co-transformed with linear sgRNA constructs (agdA-sgRNA1 and agdA-sgRNA2) and Cas9 expression cassette into the protoplasts of *A. niger* D353.8. Two DNA double-strand breaks (DSBs) at the flanking sequences of the *agdA* gene were

generated by the Cas9 under the guide of two sgRNAs, and then were repaired by HR with the integration of donor DNAs. (B) Diagnostic PCR analysis of primary transformants with fluorescence of constructs YDD2 to YDD7 expressing *mCherry-pyrG* controlled by six constitutive promoters. When the donor DNAs harboring P*goi*::*mCherry-pyrG* expressing cassettes were correctly integrated into the *agdA* locus, the expected sizes of PCR products of YDD2 to YDD7 were 2749-bp, 1926-bp, 2169-bp, 1997-bp, 2110-bp and 1992-bp, with the primers of agdA-g-F/mCherry-R, respectively. The parent strain D353.8 was used as negative control, which was represented as "NC" in each electrophoretogram. For each promoter, four positive transformants were randomly selected for further quantitative fluorescence analysis via flow cytometry. (C) Representative fluorescence images in hypha from spore germination and mycelial pellets of constructs expressing *mCherry-pyrG* controlled by six constitutive promoters.

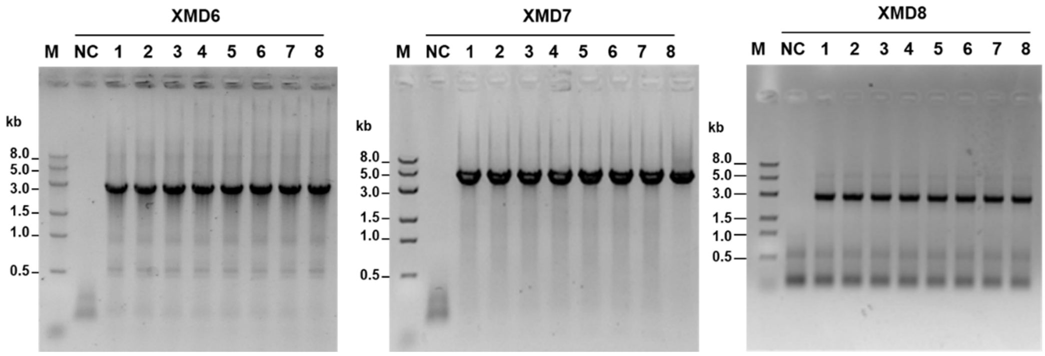

Figure S3. Construction of *A. niger* strains expressing *cexA* under the control of the P*gpdA* promoter.

Diagnostic PCR analysis of *cexA* over-expression transformants. When the donor DNAs containing P*gpdAd*::*CexA*, P*gpdAg*::*CexA* and P*gpdAg-775*::*CexA* expressing cassettes were correctly integrated into the *agdA* locus, the expected sizes of PCR products of XMD6, XMD7 and XMD8 were 2447-bp, 3647-bp and 2514-bp, with the primers of agdA-g-F/cexA-R, respectively. The parent strain D353.8 was used as negative control, which was represented as "NC" in each electrophoretogram. Positive transformants XMD6.1, XMD6.2, XMD7.1, XMD7.2, XMD8.1 and XMD8.2 were selected for further citric acid fermentation.

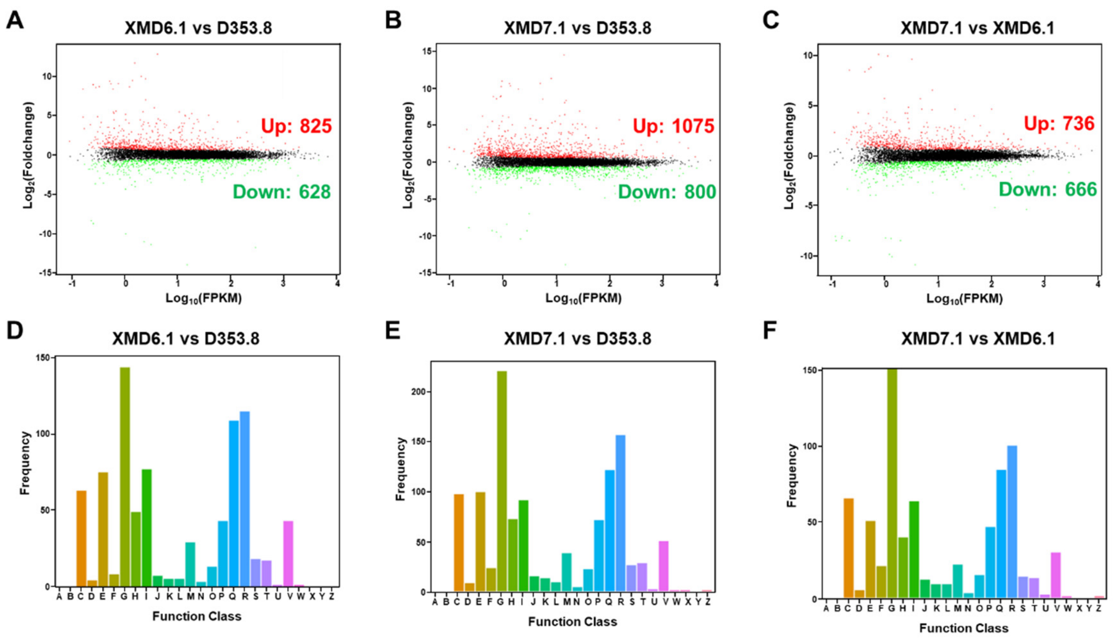

Figure S4. Comparative transcriptome analysis of *cexA* expressing constructs in submerged citric acid fermentation.

(A-C) Bland–Altman plot (M-versus-A plot, MA plot) of differentially expressed genes among D353.8, XMD6.1 and XMD7.1. Each dot represents a single gene. The dots colored in red and green stand for significant up-regulated and down-regulated genes, respectively. Black dots stand for the genes without significant difference in expression among three samples. The numbers in red and green represent the total number of significantly up-regulated and down-regulated genes, respectively. (D-F) Summary of COG classifications on differentially expressed genes among D353.8, XMD6.1 and XMD7.1. The frequency in y-axis reflects the enrichment of functional classification of differentially expressed genes in in different experimental groups. Function classes A-Z in x-axis represented the different physiological functions. A: RNA processing and modification; B: Chromatin structure and dynamics; C: Energy production and conversion; D: Cell cycle control, cell division, chromosome

partitioning; E: Amino acid transport and metabolism; F: Nucleotide transport and metabolism; G: Carbonhydrate transport and metabolism; H: Coenzyme transport and metabolism; I: Lipid transport and metabolism; J: Translation, ribosomal structure and biogenesis; K: Transcription; L: Replicatin, recombination and repair; M: Cell wall/membrane/envelope biogenesis; N: Cell motility; O: Posttranslational modification, proten turnover, chaperones; P: Inorganic ion transport and metabolism; Q: Secondary metabolites biosynthesis, transport and catabolism; R: General function prediction only; S: Function unknown; T: Signal transduction mechanisms; U: Intracellular trafficking, secretion, and vesicular transport; V: Defense mechanisms; W: Extracellular structures; X: Mobilome prophages, transposons; Y: Nuclear structure; Z: Cytoskeleton.

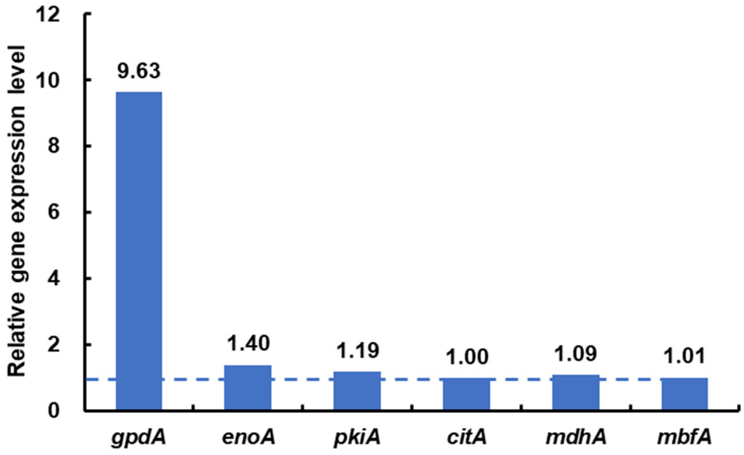

Figure S5. Relative transcription level of genes of the analysed promoters in *A.niger* D353.8.

The transcription level of each gene was normalized to the expression value of *CitA*, which was arbitrarily set to 1.

Table S1. Strains and plasmids used in the study.

Name	Genotype	Reference
**strains**		
D353.8	*kusA::hph, pyrG::hph, hyg*^{R}	[1]
YDD1	*kusA::hph, pyrG::hph, agdA::*P*gpdAd-mCherry-PyrG, hyg*^{R}	This study
YDD2	*kusA::hph, pyrG::hph, agdA::*P*gpdAg-mCherry-PyrG, hyg*^{R}	This study
YDD3	*kusA::hph, pyrG::hph, agdA::*P*enoA-mCherry-PyrG, hyg*^{R}	This study
YDD4	*KusA::hph, pyrG::hph, agdA::*P*pkiA-mCherry-pyrG, hyg*^{R}	This study
YDD5	*KusA::hph, pyrG::hph, agdA::*P*citA-mCherry-pyrG, hyg*^{R}	This study
YDD6	*KusA::hph, pyrG::hph, agdA::*P*mdhA-mCherry-pyrG, hyg*^{R}	This study
YDD7	*KusA::hph, pyrG::hph, agdA::*P*mbfA-mCherry-pyrG, hyg*^{R}	This study
XMD6	*KusA::hph, pyrG::hph, agdA::*P*gpdAd-cexA, pyrG*$^+$*, hyg*^{R}	This study
XMD7	*KusA::hph, pyrG::hph, agdA::*P*gpdAg-cexA, pyrG*$^+$*, hyg*^{R}	This study
XMD8	*KusA::hph, pyrG::hph, agdA::*P*gpdAg-775-cexA, pyrG*$^+$*, hyg*^{R}	This study
**Plasmids**		
pSM-AnpyrG	P*trpC*::*AnpyrG*::T*trpC*	[1]
pCas9-AnpyrG	P*glaA*::nls-*Cas9*-nls::T*glaA, pyrG*^{R}	[1]
psgRNA6.1	P*5S rRNA*::sgRNA::T*poly(T)*$_6$	[2]
psgRNA6.18	P*5S rRNA*::agdA-sgRNA1::T*poly(T)*$_6$	This study
psgRNA6.19	P*5S rRNA*::agdA-sgRNA2::T*poly(T)*$_6$	This study
pFPSM	*mCherry-pyrG*::T*trpC*	This study
pYDD1	P*gpdAd*::*mcherry-pyrG*::T*trpC*	This study

pYDD2	P*gpdAg*::*mcherry-pyrG*::T*trpC*	This study
pYDD3	P*enoA*::*mcherry-pyrG*::T*trpC*	This study
pYDD4	P*pkiA*::*mcherry-pyrG*::T*trpC*	This study
pYDD5	P*citA*::*mcherry-pyrG*::T*trpC*	This study
pYDD6	P*mdhA*::*mcherry-pyrG*::T*trpC*	This study
pYDD7	P*mbfA*::*mcherry-pyrG*::T*trpC*	This study
pXMD6	P*gpdAd*::*cexA*::T*trpC*	This study
pXMD7	P*gpdAg*::*cexA*::T*trpC*	This study
pXMD8	P*gpdAg-775::cexA*::T*trpC*	This study

Table S2. Protospacers used in this study.

Name	Protospacer Sequence (5' to 3') [a]	PAM
AgdA-sgRNA1	ACTTCACCATGGCTCGCTTA	AGG
AgdA-sgRNA2	ATGGGCGGAAAACTGGGTAT	TGG

Note: [a], sgRNA target sequences were predicted by a software package sgRNAcas9[1] (https://sourceforge.net/projects/sgrnacas9/). sgRNAcas9 enables the fast design of sgRNA target sequences with minimized off-target effects by predicting the genome-wide Cas9 potential off-target cleavage sites (POT). The criteria for selection of efficient sgRNAs were instituted: (1) G/C content between 40% and 60%; (2) no more than 1 POT sites; (3) no more than 4 continuous T nucleotides (4,6 nucleotide poly (T) tract acts as a termination signal for RNA pol III), or other homopolymer sequences (more than 5 continuous A or C or G, more than 6 dinucleotide or trinucleotide repeats); (4) no *Bbs*I restriction site.

Table S3. Primers used in this study.

Primer name	Primer Sequence (5' to 3')
Primers to construct *mCherry-pyrG* fused reporter plasmids	
mCherry-F	caggaaacagctatgacgctagcgacgtcgtttaaacttaattaacatATGGTGAGCAAGGGCGAGGAG
mCherry-R	aggtgggacttcgaagacatggaaccgccaccgccggatccgccaccgccTCGCGACTTGTACAGCTC
pSM-AnpyrG-Frev	gatccggcggtggcggttccATGTCTTCGAAGTCCCAC
pSM-AnpyrG-Rrev	GGTCATAGCTGTTTCCTGTGTG
PgpdAd-F	gaaacagctatgacgctagcCGGAGAATATGGAGCTTCATCG
PgpdAd-R	gacgtcttaattaagtttaaacGGTGATGTCTGCTCAAGCG
PgpdAg-F	gaaacagctatgacgctagcCTCAGGAGGCGAATAGATAA
PgpdAg-R	gacgtcttaattaagtttaaacTGTCTATGTGGCGGGGTAAT
PenoA-F	gaaacagctatgacgctagcCGGATTGAGTTGTGTGGAAA
PenoA-R	gacgtcttaattaagtttaaacCTGGAGGGGGATGAGTTATG
PpkiA-F	gaaacagctatgacgctagcTCTACCTTGTGGGTGTCCAA
PpkiA-R	gacgtcttaattaagtttaaacTGACGGATGATTGATCTCTACTG
PcitA-F	gaaacagctatgacgctagcGGTAACCGATTTTCTACACAAC
PcitA-R	gacgtcttaattaagtttaaacGACCGTAAGATACAGCCGCCCA
PmdhA-F	gaaacagctatgacgctagcGCCATGTTTTGGTAGGCTGT
PmdhA-R	gacgtcttaattaagtttaaacGGTGAAATTTGGGATTGTGA
PmbfA-F	gaaacagctatgacgctagcGAGGGCGAACTGTAGCTCCT

PmbfA-R	gacgtcttaattaagtttaaacTGGATGAGAAGTCGGTGATG
Primers to construct over-expression plasmids	
cexA-F	gtttaaacttaattaagacgtcATGTCTTCAACCACGTCTTCATC
cexA-R	gccttagcatgcgaagatctCTAGTTGCCGTTGGCTTTGG
pYDD2-Frev	cgttgaagaaaaccctggccctcatATGGTGAGCAAGGGCGAGGAG
pYDD2-Rrev	gacgtcttaattaagtttaaacTGTCTATGTGGCGGGGTAATG
Primers to construct targeting sgRNA plasmids	
agdA-sgRNA1-F	caccACTTCACCATGGCTCGCTTA
agdA-sgRNA1-R	aaacTAAGCGAGCCATGGTGAAGT
agdA-sgRNA2-F	caccATGGGCGGAAAACTGGGTAT
agdA-sgRNA2-R	aaacATACCCAGTTTTCCGCCCAT
M13F	TGTAAAACGACGGCCAGT
M13R	CAGGAAACAGCTATGACC
Primers to construct donor DNAs with micro-homologue arms	
MH-agdA-sgRNA1-F	tccattctcatcaaggattacgcgaccgtgcctcgagcctCACAGGAAACAGCTATGAC
MH-agdA-sgRNA2-R	gctgtacgcgcacacctggcttgtggctgacactaccattAACCCAGGGGCTGGTGACGG
MH-agdA-sgRNA1-F-PgpdAg1011	tccattctcatcaaggattacgcgaccgtgcctcgagcctGGCCAGAATAATAAGAATG
sgRNA1-F-	tccattctcatcaaggattacgcgaccgtgcctcgagcctGTTACTTCCATTACATCATC

PgpdAg775	
MH-agdA-sgRNA1-F-PgpdAg531	tccattctcatcaaggattacgcgaccgtgcctcgagcctAGCAGGAGATCCAAATATCG
sgRNA1-F-PgpdAg319	tccattctcatcaaggattacgcgaccgtgcctcgagcctGCGGAGGACCCAGTAGTAAG
MH-agdA-sgRNA1-F-PgpdAg157	tccattctcatcaaggattacgcgaccgtgcctcgagcctCTCTTCCCTTCATCCAATTC
Primers for diagnostic PCR and qRT-PCR of transformants	
gpdA-q-F	ATGCTGTACGTTCTGCCCTC
gpdA-q-R	CTCGATGGTGCCCTTGAACT
mCherryA-q-F	ATGCGGTTCAAGGTGCACAT
mCherryA-q-R	CCTTGGTCACCTTCAGCTTG

Notes: Restriction sites are underlined. Fm represents forward primer with modification and Rm represents reverse primer with modification. The modified additional sequences were represented in lowercase letters.

Table S4. Promoter strength evaluation by flow cytometry analysis.

Strains	Promoter	Reference	Gene ID	Length (bp)	Function Description	Mean mCherry intensity value[1] (n=4)	Foldchange to P*gpdAd* [2]	*p-value* [3]
D353.8	/		/	/	/	36.49	/	/
YDD1.13	P*gpdAd*	[3]	AN8041	680	Glyceraldehyde-3-phosphate dehydrogenase	143.87±7.16	/	/
YDD2. 4	P*gpdAg*	\	An16g01830	1889	Glyceraldehyde-3-phosphate dehydrogenase	280.99±11.08	2.28	6.30E-06
YDD3.1	P*enoA*	\	An18g06250	1056	Enolase	80.55±1.29	0.41	2.94E-04
YDD4.3	P*pkiA*	[4]	An07g08990	1299	Pyruvate kinase	95.18±1.69	0.55	4.67E-04
YDD5.2	P*citA*	[5]	An09g06680	1127	Citrate synthase	75.71±1.38	0.37	2.16E-04
YDD6.1	P*mdhA*	[6]	An15g00070	1240	Malate dehydrogenase	82.44±1.17	0.43	3.78E-04

YDD7.1	P*mbfA*	[6]	An02g12390	1122	Transcription coactivator	80.53±2.15	0.41	1.56E-04

[1] For each construct, the mCherry intensity of 100,000 conidia were analyzed by flow cytometry. [2] Foldchange to P*gpdAd* was calculated by normalized mean mCherry intensity value of each promoter divided by normalized mean mCherry intensity value of P*gpdAd*. The normalized mean mCherry intensity value of each promoter was obtained by minus the background mean fluorescence intensity value of D353.8.[3] Pairwise Student 's *t-test* was conducted to compare constructs YDD2 to YDD7 controlled by native promoters to the YDD1.13 with P*gpdAd* from *A. nidulans*.

Table S5. Summary of RNA sequencing and mapping in this study.

Samples	Clean reads	GC Content	Q30	Total Mapped Reads	Uniquely Mapped Reads	Multiple Map Reads
D353.8	58,213,738	54.28%	94.63%	55,718,717 (95.71%)	55,415,183 (95.19%)	303,534 (0.52%)
XMD6.1	43,036,282	53.99%	94.30%	40,835,059 (94.89%)	40,585,249 (94.30%)	249,810 (0.58%)
XMD7.1	42,728,650	54.06%	94.65%	38,483,204 (90.06%)	38,247,221 (89.51%)	235,983 (0.55%)

References

1. Zhang, L.H.; Zheng, X.M.; Cairns, T.C.; Zhang, Z.D.; Wang, D.P.; Zheng, P.; Sun, J.B. Disruption or reduced expression of the orotidine-5 '-decarboxylase gene *pyrG* increases citric acid production: a new discovery during recyclable genome editing in *Aspergillus niger*. *Microb. Cell. Fact.* **2020**, *19*.
2. Zheng, X.; Zheng, P.; Zhang, K.; Cairns, T.C.; Meyer, V.; Sun, J.; Ma, Y. 5S rRNA Promoter for Guide RNA Expression Enabled Highly Efficient CRISPR/Cas9 Genome Editing in *Aspergillus niger*. *ACS Synth. Biol.* **2019**, *8*,1568-1574.
3. Punt, P.J.; Dingemanse, M.A.; Kuyvenhoven, A.; Soede, R.D.; Pouwels, P.H.; van den Hondel, C.A. Functional elements in the promoter region of the *Aspergillus nidulans gpdA* gene encoding glyceraldehyde-3-phosphate dehydrogenase. *Gene.* **1990**, *93*,101-109.
4. Storms, R.; Zheng, Y.; Li, H.; Sillaots, S.; Martinez-Perez, A.; Tsang, A. Plasmid vectors for protein production, gene expression and molecular manipulations in *Aspergillus niger*. Plasmid. **2005**, *53*, 191-204.
5. Dave, K.; Punekar, N.S. Utility of *Aspergillus niger* citrate synthase promoter for heterologous expression. *J. Biotechnol.* **2011**, *155*,173-177.
6. Blumhoff, M.; Steiger, M.G.; Marx, H.; Mattanovich, D.; Sauer, M. Six novel constitutive promoters for metabolic engineering of *Aspergillus niger*. Appl. Microbiol. Biotechnol. 2013, 97,259-267.
